# The role of weaning in brace treatment for developmental dysplasia of the hip: time to define best practice?

**DOI:** 10.1302/2633-1462.66.BJO-2025-0053.R1

**Published:** 2025-06-11

**Authors:** Joanna Craven, Ioan Davies, Daniel C Perry

**Affiliations:** 1 University of Liverpool, Liverpool, UK; 2 Wales Deanery, Cardiff, UK; 3 Cardiff University, Cardiff, UK; 4 Alder Hey Children’s Hospital, Liverpool, UK

**Keywords:** DDH, Weaning, brace treatment, Developmental dysplasia of the hip (DDH), Clinicians, randomized controlled trials, Orthopaedic Surgery, hips, paediatric orthopaedic surgeons, clinical studies, residual acetabular dysplasia, clinical outcomes

## Abstract

**Aims:**

In infants aged under six months with developmental dysplasia of the hip (DDH), the use of a removable brace is considered the gold-standard treatment. However, considerable variation exists for brace removal after ‘successful’ treatment. Some clinicians support immediate cessation, while others prefer weaning of the brace. This study aimed to explore clinicians’ understanding of weaning, and to identify current practices and the rationale behind different approaches, in order to inform the design of a future randomized controlled trial (RCT).

**Methods:**

A survey was developed using Google Forms and disseminated via professional networks, social media, and the British Society of Children’s Orthopaedic Surgery mailing list. It targeted clinicians involved in DDH care, gathering information on demographics, treatment protocols, criteria for removal, and weaning practices. Quantitative and qualitative data were analyzed to identify patterns and variability.

**Results:**

In total, 139 clinicians from 25 countries responded, with 50% from the UK. Most respondents (87.8%) followed a protocol for brace treatment, with considerable variation in definition and implementation of weaning. ‘Weaning’ was most commonly defined as a gradual reduction in brace wear over time (n = 103, 74.1%). Overall, 47.4% of respondents (n = 65) reported never weaning, 39.4% (n = 54) always wean, and 13.1% (n = 18) varied their approach. Among clinicians who always wean, the most common approach involved gradually reducing the hours per day over several weeks (n = 28, 51.9%). However, for those who sometimes wean, the most frequent practice was night-time only wear (n = 8, 44.4%). Durations of weaning differed, although the majority of clinicians reported weaning periods from two to six weeks. There is broad support for a future RCT, with 75.9% (n = 105) expressing a willingness to participate.

**Conclusion:**

This survey highlights considerable variability in weaning practices for brace treatment in DDH, and underscores the need for standardized terminology and protocols. These findings provide a foundation for designing a RCT to evaluate weaning compared with immediate brace cessation, ultimately informing evidence-based guidelines.

Cite this article: *Bone Jt Open* 2025;6(6):685–690.

## Introduction

Developmental dysplasia of the hip (DDH) affects approximately 1% of newborns, with the most severe form, complete dislocationoccurring in one in 1,000 infants.^1-3^ For infants aged under six months with reducible hips, treatment with a removable brace is the gold standard, achieving success in 90% of cases within the first three months of life.^4,5^ However, clinical practice varies considerably when it comes to removing the brace following ‘successful’ treatment.^6^ While some clinicians advocate for immediate cessation, others favour a gradual ‘weaning’ process, allowing additional time for hip development.

The current evidence base is limited, with no randomized controlled trials (RCTs) comparing weaning to immediate cessation.^7^ The only two available comparative studies are retrospective and non-randomized, with relatively small sample sizes, limiting the strength of their conclusions.^8,9^ Together, these studies indicate that while various discontinuation strategies are used in clinical practice, there is currently no strong evidence to favour weaning over immediate cessation.

This lack of high-quality data has resulted in clinical equipoise and substantial variation in practice. Surveys indicate that most clinicians in the USA and Europe implement some form of weaning, while in the UK practices are divided, with 65% favouring immediate removal and 35% adopting a weaning approach.^6,8^ Advocates of weaning suggest it may reduce the risk of residual acetabular dysplasia and long-term complications such as early-onset arthritis, while opponents argue that it adds an unnecessary burden without clear benefit.^8,10,11^

Consensus exercises have produced mixed results. The International Hip Dysplasia Institute (IHDI) supports weaning with night-time use only,^12^ whereas the British Society of Children’s Orthopaedic Surgery (BSCOS) has not reached a consensus.^13^

Adding to this complexity, there is a lack of standardized terminology to describe what ‘weaning’ entails. In clinical practice, it is critical that terms are clearly defined and universally understood to ensure consistency in communication, research, and patient care.^14^ Without agreement on the meaning of ‘weaning’, it is challenging to compare outcomes, standardize treatment pathways, or design robust clinical studies.

To address these uncertainties and lay the groundwork for a future RCT, we conducted a survey of clinicians to better understand weaning practices. This study seeks to define what ‘weaning’ means, identify when and how clinicians initiate brace removal, and explore the underlying rationale for their approaches. By elucidating these practices and promoting standardization of language, we aim to inform the design of a robust clinical trial and ultimately provide evidence to guide standardized treatment pathways in DDH management.

## Methods

A survey was developed using Google Forms (Google, USA) (Supplementary Material) by the lead and senior authors (JC, DCP). It was disseminated through professional networks and social media targeting clinicians involved in DDH care, and was also shared via the mailing list of the BSCOS. The survey remained open for a period of four months (September 2024 to January 2025).

The survey collected information on participants’ country of practice, professional role, and the number of DDH cases they manage annually. Participants were asked to define ‘weaning’ in a free-text box, allowing for interpretations of the term. Subsequent questions explored the use of protocols to guide brace treatment for DDH, the origin of these protocols, the criteria clinicians use to decide when to remove or begin the process of removing the brace, and whether a weaning regime is employed. For those using a weaning regime, details were gathered on the timing and duration of brace wear during the regime, as well as the rationale for their clinical practice. Finally, participants were asked if they would be willing to participate in a future RCT and to provide input on what they believe the weaning intervention should entail.

Free-text responses were independently categorized into domains by two reviewers (JC, ID), who subsequently discussed and reached consensus on the final domain classifications.

## Results

The survey collected 139 responses from individuals across 25 countries (Table I), with half of the participants located in the UK. Responses spanned six of the seven continents. Eight of the participating countries were classified as low- or middle-income. The majority of respondents were paediatric orthopaedic surgeons (n = 131, 94%), with most managing at least ten cases of DDH annually (n = 115, 83%) (Table II).

**Table I.** Country of participant practice (total n = 139).

Country of participant practice and World Bank country classification by income level for 2024 to 2025^15^	N (% of group)
**High-income (n = 116, 83.5%)**	
Australia	2 (1.4)
Belgium	2 (1.4)
Canada	3 (2.2)
Czechia	1 (< 1)
Denmark	1 (< 1)
UK	70 (50.3)
France	1 (< 1)
Germany	2 (1.4)
Ireland	7 (5)
Israel	14 (10.1)
Netherlands	2 (1.4)
New Zealand	1 (< 1)
Poland	1 (< 1)
Portugal	1 (< 1)
Saudi Arabia	2 (1.4)
Spain	1 (< 1)
Sweden	1 (< 1)
USA	4 (3.1)
**Middle-income (n = 22, 15.8%)**	
Argentina	1 (< 1)
Bangladesh	1 (< 1)
Chile	1 (< 1)
India	11 (7.9)
South Africa	6 (4.3)
Turkey	1 (< 1)
Ukraine	1 (< 1)
**Low-income (n = 1, < 1%)**	
Palestine	1 (< 1)

**Table II.** Number of cases of developmental dysplasia of the hip seen per annum.

Case seen per annum	N (%)
< 10	24 (17.3)
10 to 30	58 (41.7)
> 30	57 (41)

In the context of DDH, ‘weaning’ was most commonly defined as a gradual reduction in brace wear over time, encompassing terms such as ‘gradual removal’, ‘decreasing wear over time’, and ‘transition period’. Some respondents described it as wearing the brace for less than 24 hours a day, while others referred to ‘weaning’ as any period of brace use following hip normalization. Additionally, some defined it simply as wearing the brace exclusively at night (Table III).

**Table III.** Defining ‘weaning’.

Description domain	N (%)	Direct quote
Gradual reduction description	103 (74.1)	“going from full time to part time to off completely”
Time-specific description	5 (3.6)	“less than 24/7” bracing
Threshold-specific description	6 (4.3)	“a period of time after the hip is ultrasonographically normal”
Routine-specific description	8 (5.8)	“brace only during sleeping hours”
Other	5 (3.6)	“Controlled period of increasing freedom of movement”, “when the brace isn’t worn anymore”
Unsure	12 (8.6)	

A total of 122 respondents (87.8%) reported following a protocol to guide their DDH brace treatment. The majority adhered to a departmental protocol (n = 73, 59.8%), while others followed national (n = 5, 4.1%) or personal protocols (n = 44, 36.1%). The majority of participants (n = 88, 63.3%) reported that the brace regimen varied depending on the severity of DDH.

When asked about the criteria for determining when a brace can be removed or the removal process can begin, the most common response was an α angle of > 60° (n = 65, 46.7%), followed by a fixed duration of brace treatment (n = 33, 23.7%) (Table IV).

**Table IV.** Criteria for determining when a brace can be removed/the removal process can begin.

Criteria for determining when a brace can be removed or the removal process can begin	N (%)
α angle > 60°	65 (46.7)
After a fixed period of brace treatment	33 (23.7)
‘Normal’ ultrasound Quote: “determined by a normal hip scan - based on normal angles, location/depth of the femoral head, cartilaginous”	11 (7.9)
After a fixed period of brace treatment AND α angle > 60°	10 (7.2)
A clinical decision Quote: “I don't have a fixed measure or time. It’s a mix of clinical, radiological outcome and parental understanding”	9 (6.5)
Sequential ‘normal’ ultrasound Quotes: “After two “normal” USS” “α angles > 60 on 2 separate occasions 4 weeks apart” “at least 2 scans 2 weeks apart with α angle > 60”	5 (3.6)
Acetabular index	2 (1.4)
α angle > 65°	1 (< 1)
α angle correct for age	1 (< 1)
“After 1 month if the acetabular depth is under 6 mm”	1 (< 1)
“Alpha > 60 AND femoral head coverage > 50% and clinically stable hip”	1 (< 1)

USS, ultrasound.

Regarding weaning practices, 47.4% of respondents (n = 65) reported always removing the brace immediately once treatment is deemed successful. In contrast, 39.4% (n = 54) always employ a weaning process, and 13.1% (n = 18) vary their approach based on the individual infant.

For the clinicians who always wean, 51.9% (n = 28) employ a regime of reducing hours worn per day over a number of weeks, 31.5% (n = 17) do night-time only wearing or night-time and naps (1.9%). Of the clinicians who always wean, 14.8% (n = 8) vary that regime dependent on the infant. The length of the weaning regime was between two to six weeks for 75.9% (n = 41) (Table V).

**Table V.** Weaning regime, duration, and rationale breakdown.

Variable	Always wean n = 54 (39.4%)	Sometimes wean n = 18 (13.1%)	Never wean n = 65 (47.4%)
**Regime, n (%)**			
Reducing hours worn per day over a number of weeks	28 (51.9)	7 (38.8)	-
Night-time	17 (31.5)	8 (44.4)	-
Night-time and naps	1 (1.9)	0	-
12 hours per day	0	1 (5.5)	
Weaning regime variable	8 (14.8)	2 (11.1)	-
**Duration**			
< 2 wks	1 (1.9)	1 (5.5)	-
2 to 4 wks	21 (38.8)	8 (44.4)	-
4 to 6 wks	20 (37)	5 (27.7)	-
6 to 8 wks	7 (13)	2 (11)	-
8 to 10 wks	1 (1.9)	0	-
10 to 12 wks	2 (3.7)	1 (5.5)	-
> 12 wks	2 (3.7)	1 (5.5)	-
**Rationale**			
Personal opinion	29 (40.3)		25 (38.5)
Theory	22 (30.5)		13 (20)
Evidence	8 (11.1)		29 (44.6)
Following an agreed protocol	28 (38.8)		30 (46.2)
Parent preference	6 (8.3)		8 (12.3)
Teaching and training/mentors	5 (6.9)		1 (1.5)
Avoiding disruption to the family	0		3 (4.6)

For the clinicians who sometime wean, their indications for a weaning regime included severity of DDH (n = 7, 43.8%), age of the infant (n = 4, 25%), time to normalization (n = 3, 18.8%), ultrasound characteristics (n = 1, 6.3%), and parental attitudes (n =1, 6.3%).

The clinicians who sometimes wean were split between 44.4% (n = 8) employing a regime of night-time only brace wearing and 38.8% (n = 7) reducing the hours worn per day over a number of weeks. The length of the weaning regime was between two and six weeks for 72.1% (n = 13) (Table V).

In relation to a future RCT comparing weaning with immediate cessation of brace treatment, 75.9% (n = 104) of respondents expressed a willingness to participate, 13.1% (n = 18) were undecided, and 10.9% (n = 15) indicated they would not wish to take part. Respondents preferred that the weaning process begin as soon as the hip is deemed ‘normal’, with 50% (n =67) favouring the initiation of a night-time weaning regimen. The preferred duration for this regimen was two to six weeks, also supported by 50% of respondents (n = 47).

## Discussion

The findings of this survey highlight considerable variation among clinicians, both within the UK and internationally, regarding the removal of brace treatment for DDH. This variation underscores a critical gap in high-quality evidence to guide best-practice guidelines for brace treatment in DDH. This has been recognized as a pressing issue by initiatives such as the Getting It Right First Time (GIRFT) programme and the STEPS worldwide charity.^16,17^

Efforts to achieve consensus within the BSCOS have failed to resolve variability in weaning practices, underscoring the lack of agreement. While previous surveys have asked whether a weaning regime is employed,^6,18^ this is the only study exploring the concept in greater detail, offering a deeper understanding of current practices, including what clinicians mean by weaning, whether they implement it, and their rationale for doing so.^18^

Survey results reveal that while most clinicians use the term ‘weaning’ to describe the transition period between full-time brace use and complete removal, there is considerable ambiguity and inconsistency in its definition. This lack of a clear and consistent understanding complicates communication, hinders the ability to compare clinical outcomes, and poses significant challenges in the design and interpretation of research studies. We recommend defining ‘weaning’ as a “gradual reduction in brace wearing over time, beginning once full-time brace use has ceased”. Notably, this excludes any phase of continued full-time brace use after the hip has ‘normalized’, which should instead be referred to as a ‘consolidation period’. Establishing this distinction is critical to ensure clarity in both clinical and research contexts.

The survey results reveal a lack of consensus on when brace treatment is considered ‘successful’ and when removal should begin. However, aligning with the BSCOS consensus exercise, the majority of clinicians regard a centred hip, with an α angle of 60° as the key criteria for removal.

The survey demonstrates strong support among clinicians for a future RCT comparing weaning of brace treatment to immediate cessation. RCTs are essential to address these challenges and align clinical practice with the goals of GIRFT. Designing these trials will require precise terminology and a shared understanding of key concepts, including the differentiation between total brace duration, consolidation periods, and weaning processes.

By addressing these gaps, this survey lays the groundwork for future research, providing a foundation for the development of standardized care pathways that will improve outcomes for infants with DDH and reduce the burden on families.

**Take home message**

- There is significant variation in weaning practices for brace treatment for developmental dysplasia of the hip, with no consensus on the optimal approach.

- A randomized controlled trial is needed to provide robust evidence to guide best practice and standardize care.

## Data Availability

The data that support the findings for this study are available to other researchers from the corresponding author upon reasonable request.
